# Challenges to the Programmatic Implementation of Ready to Use Infant Formula in the Post-Earthquake Response, Haiti, 2010: A Program Review

**DOI:** 10.1371/journal.pone.0084043

**Published:** 2013-12-31

**Authors:** Leisel E. Talley, Erin Boyd

**Affiliations:** 1 Emergency Response and Recovery Branch, Centers for Disease Control and Prevention, Atlanta, Georgia, United States of America; 2 Program Division, Nutrition Section, United Nations Children’s Fund, New York City, New York, United States of America; University of Alabama at Birmingham, United States of America

## Abstract

**Background and Objectives:**

Following the 2010 earthquake in Haiti, infant and young child feeding was identified as a priority nutrition intervention. A new approach to support breastfeeding mothers and distribute ready-to-use infant formula (RUIF) to infants unable to breastfeed was established. The objective of the evaluation was to assess the implementation of infant feeding programs using RUIF in displaced persons camps in Port-au-Prince, Haiti during the humanitarian response.

**Methods:**

A retrospective record review was conducted from April–July, 2010 to obtain data on infants receiving RUIF in 30 baby tents. A standardized data collection form was created based on data collected across baby tents and included: basic demographics, admission criteria, primary caretaker, feeding practices, and admission and follow-up anthropometrics.

**Main Findings:**

Orphans and abandoned infants were the most frequent enrollees (41%) in the program. While the program targeted these groups, it is unlikely that this is a true reflection of population demographics. Despite programmatic guidance, admission criteria were not consistently applied across programs. Thirty-four percent of infants were undernourished (weight for age Z score <−2) at the time of admission. Defaulting accounted for 50% of all program exits and there was no follow-up of these children. Low data quality was a significant barrier.

**Conclusions:**

The design, implementation and magnitude of the ‘baby tents’ using RUIF was novel in response to infant and young child feeding (IYCF) in emergencies and presented multiple challenges that should not be overlooked, including adherence to protocols and the adaption of emergency programs to existing programs. The implementation of IYCF programs should be closely monitored to ensure that they achieve the objectives set by the humanitarian community and national government. IYCF is an often overlooked component of emergency preparedness; however to improve response, generic protocols and pre-emergency training and preparedness should be established for humanitarian agencies.

## Introduction

Exclusive breastfeeding of infants up to six months of age provides the best nutrition and immunologic protection for the promotion of healthy growth and development in non-crisis and crisis settings. [1], [2] While numerous myths and cultural taboos surround the ability of mothers to breastfeed in the immediate aftermath of an emergency, breastfeeding is physiologically possible for most women. [1], [3] There are however, a minority of mothers in crisis settings who may have difficulty nursing their infants due to stress, fatigue, lack of privacy, dehydration, and inadequate diet. [1] Additionally, the immediate chaos and impact of the emergency may result in the death of mothers or separation of mothers and infants. Infants less than six months of age, who are not breastfed, need urgent identification and targeted skilled feeding support.

The priority approach to feeding these infants should be through relactation with their own mother or breastfeeding through a wet nurse. [1] If these options are not possible or when skilled staff, such as health providers or infant feeding counselors, indicate that breast milk substitutes (BMS) are necessary, this must be accompanied by training on hygiene, preparation and use of BMS. BMS in an emergency carries a substantial risk of malnutrition, morbidity and mortality, and should be a last resort option, used only when other safer options have been fully explored. In the majority of natural disasters and complex emergencies, BMS are donated and widely distributed with little monitoring or guidance to mothers. This often results in poor preparation using contaminated water, thereby significantly increasing infants’ risk of morbidity and mortality. [4] In emergency situations where there are large number of non-breastfed infants, the substantial risks associated with BMS distribution must be weighed against the risk malnutrition among infants consuming commercial products not intended for use as a BMS.

Haiti presented a complex set of challenges for addressing infant and young child feeding (IYCF) in the aftermath of the 2010 earthquake, including sub-optimal feeding practices prior to the earthquake as demonstrated by an exclusive breastfeeding prevalence of 21.7% in Port-au-Prince. [5] Mixed feeding, whereby mothers give breast milk and other replacement feeds, such as diluted sweetened condensed milk, was pervasive. [5] Furthermore, cultural taboos related to breastfeeding dissuaded many mothers from appropriate IYCF practices. [1], [3] In Haiti, beliefs that “strong emotions or bad news either spoil mother’s milk or stop its production; and that a mother who spends time under the sun cannot breastfeed because her milk becomes hot and may cause diarrhea to the child were particularly relevant in the post-earthquake displaced persons tents”. [6] Haiti also had the highest HIV prevalence rate (3.8%) in the Caribbean region, which served as an additional perceived barrier to breastfeeding. [5] Another complexity of the situation was that 20% of Haitian infants/children were classified as orphaned or vulnerable prior to the earthquake. [5]

Based on the large-scale displacement into makeshift shelters with significant issues of crowding; poor sanitary conditions, including limited excreta disposal; limited cooking facilities; the cessation of pre-existing weak public health services; and an increased risk for the spread of communicable diseases; the humanitarian community, in collaboration with the Ministère de la Santé Publique et de la Population (MSPP), identified the need to address IYCF, including the provision and management of BMS as a part of the nutrition response. Specifically, Ready to Use Infant Formula (RUIF) was identified as the safest option. RUIF is a commercially produced (Codex Standard 72–1981) hygienic, pre-mixed liquid infant formula that is consumed directly from the container or a cup, avoiding the use of water, bottles and teats. RUIF was already in use on an individual basis in select hospitals for the care of premature infants.

The National Nutrition Cluster (NNC) in Haiti, chaired by MSPP and UNICEF, was responsible for the procurement, management and administration of RUIF. The NNC non-governmental (NGO) partners established *Points de Conseil en Nutrition pour Bébé* (PCNB) or ‘baby tents’ in most tent cities throughout Port-au-Prince, Leogane and Jacmel. The baby tents provided a safe place for mothers to breastfeed and caregivers to receive IYCF counseling. The intent was to provide RUIF in some of these tents through strict criteria to targeted infants. [7]

## Materials and Methods

### Intervention

Baby tents were focused on providing a quiet place to breastfeed; promote and maintain breastfeeding; give psychosocial support; support relactation; and, screen for growth faltering and acute malnutrition. The baby tents adhered to the national guidelines developed by the NNC during the initial weeks following the earthquake. [7] A guideline-based compulsory training was developed and attended by all NGOs establishing baby tents and offering RUIF.

In line with the *Operational Guidance on Infant and Young Child Feeding in Emergencies*, admission criteria were defined as: infants under 12 months of age living in areas directly affected by the earthquake with no possibility of being breastfed based on one of the following situations: [1], [7]

- Mother absent or dead
- Critically ill mother
- Re-lactating mother until lactation re-established
- Infant rejected or abandoned by mother
- Mother survivor of sexual violence not wishing to breastfeed
- Infants of known HIV-infected mothers who were not breastfed prior to the earthquake and were currently exclusively fed on powdered infant formula
- Infants who were artificially fed prior to the earthquake

Infants were discharged from the program at 12 months of age or to treatment if they became acutely malnourished. The NCC ensured adequate RUIF for all infants enrolled until they reached 12 months. Enrollment of new infants into the program ceased in August, 2010.

Infants were offered the following services at the baby tents: anthropometric assessment (weight, length and MUAC screening), referral for management of severe acute malnutrition, and referral for health care. Additionally, the program guidance stated that if an infant less than 6 months weighed less than 3 kg, they should be referred to the Centre Nutritionnel Supplementaire for inpatient treatment of acute malnutrition and not receive RUIF. Caretakers were offered assessment of current feeding practices and counseling on age-appropriate feeding of the child, including optimal breastfeeding positions and attachment, expressing breast-milk, feeding frequency, and advice on moving to exclusive breastfeeding if other liquids and foods were fed to infants younger than 6 months, relactation and complementary feeding. During the feeding assessment, non-breastfed children were identified and their eligibility for RUIF determined; caretakers were then counseled and trained how to feed RUIF using the cup. Depending on the implementing NGO, caretakers returned on a daily or weekly basis to collect additional supplies of RUIF and for follow-up of the infant. The objective of this evaluation was to assess the implementation of the infant feeding programs in response to the 2010 earthquake in Haiti.

## Methods

A retrospective record review was conducted from 14 to 27 July, 2010 to obtain data on infants receiving RUIF in baby tents. Selection criteria for baby tents included: 1) the baby tent was operational in April 2010 in Port-au–Prince, 2) the baby tent was enrolling infants at the time of sample selection (June 2010), and 3) the baby tent planned to continue services through December 2010. A standardized data collection form was created based on data collected across baby tents and NGOs and included: basic demographics, admission criteria, primary caretaker, feeding practices, and admission and follow-up anthropometrics. Age of the infant was collected from the caregivers; the majority was able to provide a date of birth for the infant. Food intake was assessed through a 24 hour recall for each infant. Based on the specific NGO program, beneficiaries received RUIF either daily or weekly; therefore the records for each infant receiving RUIF differ in terms of the number of visits and anthropometric measurements.

Thirty baby tents administered by six NGOs were included in the evaluation. Trained enumerators extracted data from 590 individual infant records of infants admitted between April 1 and June 29, 2010. There were 16,946 individual visits recorded among these infants during this time period. Of the 590 infant admitted, 493 (83.6%) were included in the analysis. Infants were excluded for the following reasons: missing data (sex, date of birth, admission weight), implausible weight-for-age at admission, age was outside of the admission criteria (i.e greater than 12 months of age), enrollement start date was prior to April as defined by the protocol, or if the infant only attended the baby tent one time. The majority of exclusions (44.3%) came from infants only visiting the baby tent one time. Sixty-eight percent of infants included in the analysis were between 0 and 5 months of age at admission with a median age of 4 months. There were similar numbers of boys and girls, 249 (50.5%) and 244 (49.5%), respectively.

### Statistical Analysis

Analysis was limited to records of infants who met the following criteria: date of birth recorded; sex recorded; younger than 12 months of age at enrollment; more than one visit to the baby tent recorded; weight recorded at admission and at least one subsequent visit; and plausible weight-for-age at admission. NGOs did not consistently measure length at admission or subsequent visits. The majority of measurements were rounded to 0.0 cm or 0.5 cm, therefore reliable weight-for-length and length-for-age were not assessed. Weight gain was assessed based on the difference between two weights divided by the number of days between the weights. The weight gain by age was assessed using the approximate daily weight gain by age in the *Nelson Textbook of Pediatrics*, however it was not possible to assess weight gain of infants as a result of low data quality. [8]

Data were entered into Epi Info version 3.5 (Centers for Disease Control and Prevention, Atlanta, GA, USA) and analysis was completed in SAS version 9.3 (SAS Institute Inc., Cary, NC, USA). [9], [10]Weight-for-age Z scores (WAZ) based on WHO Growth Standards were calculated in Emergency Nutrition Assessment software version Delta and the range of plausible values were set between <−6 WAZ >5.(11) Mean WAZ and standard deviations were calculated for age groups of 0–5 and 6–11 months and orphaned/non-orphaned infants at time of admission. The difference in mean WAZ between groups was tested for statistical difference using a *t-test* for comparing two group means. A *P*<0.05 was considered significant.

### Ethics Statement

The study was reviewed and approved by the Division of Nutrition, MSPP. The Institutional Review Board of the Centers for Disease Control and Prevention determined that the activity was not human subjects’ research and that the primary intent was public health practice. Caregivers provided verbal consent to participate in the baby tent program. Enumerators not involved in the implementation of the program collected data from infant cards and data collection did not involve interaction with beneficiaries. Personal identifiers were deleted from the datasets once data were entered and cleaned.

## Results

Infants were admitted for varying reasons. The most frequent reason was the infant was orphaned (41%), followed by HIV-infected mother (15%) (Table 1). Among orphans, 18.3% were single orphans where the mother had died, 81.7% were double orphans. A smaller proportion of children were reported to be abandoned (8.3%), with the majority (83%) reported to be abandoned by both parents. Based on the admission data, 49.3% of infants receiving RUIF were orphaned or abandoned and could be considered extremely vulnerable. The majority (96%) of infants with HIV-infected mother were admitted to one NGO program that focused on HIV care, which had been established and was providing BMS prior to the earthquake. However, only 14% of these infants were born before the earthquake and therefore admission of the majority of infants based on this criterion was contrary to the guidance provided in the baby tent guidelines as they were not artificially fed prior to the earthquake. A second NGO was focused on orphaned and abandoned infants; 83.4% of their admissions were based on this criterion and only 1 mother was reported to be a caregiver in this program. While re-lactation was a goal of the PCNB program, only 1% of mothers were actively attempting re-lactation.

**Table 1:** Recorded Criteria for Admission into RUIF Program among Infants, April–June 2010.

Admission Criteria	Number (%) N = 493
Orphaned	202 (41.0)
Single maternal orphan	37 (18.3)
Double orphan	165 (81.7)
Abandoned	41 (8.3)
Single maternal abandonment	7 (17.1)
Double abandonment	34 (82.9)
Mother ceased breastfeeding post-earthquake, trying re-lactation	5 (1.0)
Infant was never breastfed before the earthquake	16 (3.2)
Mother experiencing difficulty breastfeeding	68 (5.1)
Mother has an unconfirmed psychological condition	25 (5.1)
Contraindicated due to maternal medical condition or prescribed medicine	34 (6.0)
HIV-infected mother	74 (15.0)
Other	28 (5.7)

Forty-eight (9.7%) of infants in the program were erroneously admitted because they weighed less than 3 kg at younger than 6 months of age and, according to program guidelines, should have been referred for inpatient treatment of acute malnutrition. None of these infants were referred for treatment; four defaulted.

Mothers were the most frequent (38%) caregiver present at the time of the infants’ admissions, followed by aunts and grandmothers (26% and 13%, respectively). Of note, 11% of caregivers were male: 10% fathers and 1% uncles (Table 2).

**Table 2:** Primary Caregiver of Infants Enrolled in RUIF Program, April–June 2010.

Primary Caregiver of Infant	Number (%)
	N = 493
Mother	188 (38.1)
Father	51 (10.3)
Aunt	128 (26.0)
Uncle	5 (1.0)
Grandmother	66 (13.4)
Sibling of infant	12 (2.4)
Cousin	18 (3.6)
Neighbor	8 (1.6)
Other	17 (3.4)

Forty-five infants, all less than 6 months of age, only received breast milk prior to admission; however, 86.7% of them were admitted within 1 week of their birth into the NGO program specific for infants with HIV-infected mothers. The majority of infants under 6 months of age (87%, 292/337) were receiving foods in addition to or in replacement of breast milk at the time of admission. All children aged 6 months and older were consuming food other than breast milk. Across age groups, the use of powdered infant formula and powdered milk (not intended for infant feeding) was quite high (Table 3).

**Table 3:** Results of 24 Hour Recall of Types of Food Consumed by Non-exclusively Breastfed Infants Admitted into the RUIF Program, April–June 2010*.

Food type	Infants 0–5 months	Infants 6–11 months	All ages
	Number (%)	Number (%)	Number (%)
	N = 292	N = 156	N = 448
Powdered Infant Formula	77 (26.4)	57 (36.5)	134 (29.9)
Liquid Infant Formula	29 (9.9)	4 (2.6)	33 (7.7)
Powdered Milk	70 (24.0)	42 (26.9)	112 (25.0)
Condensed Milk	21 (7.2)	10 (6.4)	31 (6.9)
Complementary Foods (semi-solids, solids,fruits, vegetables, meat )	165 (56.5)	137 (87.8)	302 (67.4)
Sugar Water	66 (22.6)	29 (18.6)	95 (21.2)
Other Liquids (Milk, Juice, Water)	17 (5.8)	10 (6.4)	27 (6.0)
Other	3 (1.0)	10 (6.4)	13 (2.9)

*Columns do not add to 100% as infants could consume more than one food item.*

WAZ were calculated as the primary anthropometric indicator of nutritional status. At admission, the mean WAZ of all infants was −1.41 (SD 1.82). Younger infants had a significantly lower WAZ upon admission compared with older infants, −1.80 versus −1.23 (p = 0.001), respectively. Among orphans, the mean WAZ at admission was −1.37 (SD 1.82). There was a significant difference in the mean WAZ at admission between NGO’s (ANOVA, p<0.001), with one NGO having a mean WAZ of −2.31.

There was no significant difference in mean WAZ (pooled *t test, t* = 0.55, p = 0.58) nor in the prevalence of underweight (χ^2^ = 0.26, p = 0.6; 50.9% and 49.1% were underweight) of orphans compared with non-orphans at admission, respectively.

Thirty-four percent of infants were underweight at enrollment; 19.5% of infants enrolled in the program were severely underweight. Of note, more infants were severely underweight (WAZ <−3) compared with moderately underweight (WAZ >−3 to <−2) (Table 4). Within one NGO program, 60% of admissions were underweight (WAZ <−2).

**Table 4:** Weight-for-Age Status of Selected Infants at Admission into the RUIF Program: Normal, Global Underweight and Severe Underweight.

	0–5 months	6–11 months	All ages
	N = 337	N = 156	N = 493
WAZ > = −2	235 (69.7)	89 (57.1)	324 (65.7)
WAZ ≥−3 to <−2	41 (12.2)	32 (20.5)	73 (14.8)
WAZ <−3	61 (18.1)	35 (22.4)	96 (19.5)

Seventy-four (15%) infants exited the program before the end of June; 27% of exits occurred among children who “aged” out of the programs at 12 months of age and 6.0% of exits were a result of death. Defaulting accounted for the majority of exits, 36 (49%). There was no further information on defaulters or deaths. Only two infants exited to breastfeeding programs through successful re-lactation programming.

The number of visits each infant made to the PCNB varied significantly, ranging from 2 to 102 visits (mean 30). Measurements were not taken at each visit; the average number of visits with a measurement was 14 (range 2–99). Older infants (6–12 months) made more visits to the baby tents (median 25.5) than younger infants (median 17) (pooled *t-test* p = 0.005).

## Discussion

### Limitations

Data quality was a significant issue, which substantially reduced the number of infants included in the analysis as well as data available for analysis. First, length was not systematically recorded at admission and the majority of recorded lengths were rounded to 0.0 cm or 0.5 cm. This is most likely due to the 0.5 cm graduation of increments on the measuring mats. Therefore, weight-for-length and length-for-age could not be calculated. Weight was assessed and recorded at admission. Subsequent weights were recorded, however almost half of the weights recorded were out of range for age appropriate weight gain for the child’s age. This may have been a result of the use of Salter scales or electronic baby scales and failure to recalibrate the scale at frequent intervals. Weight gain could not be assessed.

Accurate age data are often difficult to gather, especially for orphaned and abandoned infants. For a small number of infants either age was not recorded or it could not be reconciled. Older infants may have frequented the baby tents more often than younger infants simply because they had more opportunities to attend based on their age. Finally, there were a number of infants who were erroneously enrolled; specifically, they were older than 12 months of age at admission or were severely underweight.

### Main Discussion

The sheer scale of the disaster immediately following the earthquake in Haiti presented numerous obstacles to mounting a rapid and robust humanitarian response across all sectors. The additional complexity of large numbers of orphaned and abandoned children unable to breastfeed demanded a novel approach to IYCF. With no precedent and limited programmatic experience on the scale of the emergency presented by the Haiti earthquake, the baby tent program was designed in real time in the initial weeks following the earthquake. Within two days of the earthquake IYCF was identified as an urgent issue by UNICEF and the NCC. A rapid assessment followed which confirmed that powdered infant formula was not a viable, feasible or safe option for the response. A week and a half after the earthquake, supplies had been procured and distribution commenced. By February, an IFE specialist had been hired for UNICEF and over 50 tents were running and routinely providing RUIF. In a successful undertaking by the humanitarian nutrition community (NGOs, UN agencies and donors), 8,787 infants received RUIF in 89 baby tents during the response.

Designing a new programmatic approach to IYCF in the midst of an emergency poses many challenges. A balance must be struck between providing services while maintaining quality of the program and adhering to the program guidance. NGOs appeared to have difficulty adapting the emergency baby tent program to their existing programs and mandate. Admission criteria were altered to meet the objectives of the specific NGOs; for example, admitting all infants of HIV-infected mothers regardless of the feeding status of the infant prior to the earthquake or only admitting orphans. Additionally, NGOs did not abide by some of the programmatic policies due to inexperience with IYCF in emergencies, as well as logistics constraints to even physically access and staff the baby tents (data not presented). One of the challenges in the aftermath of the earthquake was transport of referrals. The location of inpatient treatment services was mainly in hospitals that were sometimes difficult to access, overcrowded and poorly functioning.

There must also be a balance between providing services and collecting data on individual infants. Discrepancies in data, specifically weight measurements, question the use of data to monitor an infant’s progress. A key objective of ICYF is to support and promote ideal feeding practices to achieve optimal growth and development. In addition, the lack of referral of low-weight infants (less than 3 kg) at the time of enrollment highlights the limited use of weight data to monitor an infants’ overall health. Critical attention must be given to the growth trends of the infant receiving RUIF or any other service in the baby tent, particularly for the 20% of infants who were severely underweight at the time of admission. If this cannot be achieved within the baby tent itself, it is crucial that this program be linked to a growth monitoring program and/or programs for the management of acute malnutrition in infants. This type of robust monitoring also underscores the need for skilled staff and accurate scales and measuring mats, particularly for those infants less than 6 months of age. Additionally, basic interpretation of data is needed. Sixty percent of admissions for one NGO were underweight and 35% were severely underweight. This is highly unusual and requires further investigation if this is a true anomaly reflecting varying socio-economic status within Port-au-Prince or simply poor data quality. The proportion of severe underweight to moderate underweight infants was skewed. Typically, there are more moderate cases than severe. [11] While a critical assessment of weight data is essential, the accuracy of anthropometric measurements is critical. Salter scales, as used in many baby tents, are not sufficient to detect the small changes in infant weight. A 2009 review of IYCF programs highlighted the need for improved scales, either balance beam scales or infant bench scales. [12]

Older infants (6 to 12 months of age) came more frequently to the baby tents compared with the younger infants. While frequent visitation and the chance to interact with caregivers are beneficial at any age, it is particularly beneficial for the youngest infants. This age group is particularly vulnerable when consuming RUIF and should be closely monitored and provided clear messaging on appropriate infant feeding. Additionally, 11% of caregivers were male. This is an important programmatic consideration when designing programs and messaging on feeding as these are primarily targeted towards mothers and women.

The proportion of infants reported to be orphaned or abandoned (49%) was exceedingly high. Prior to the earthquake, 20% of children were in this category, which indicates that there was a pre-existing problem in Haiti linked to abandonment. [5] While an objective of the baby tent program was to target and protect orphaned/abandoned infants, it is unlikely that this increase in the percentage of orphaned/abandoned infants is a true reflection of the prevalence of these children in the population. Fifty percent of exits from the program were defaulters. There was no recorded follow-up of these infants to determine the reason for defaulting. The ability to trace these extremely vulnerable children is critical and, while it was incorporated into the guidance, many families left the tent cities at some point, making follow up impossible. This is a significant lapse in the programmatic response.

## Conclusions

Responding to infant feeding needs in emergency contexts continues to present a challenge. Since 2010, RUIF has not been used on a large scale as subsequent emergencies have not presented the same context as Haiti. The floods in Pakistan in 2010, the Horn of Africa crisis of 2011, the Sahel emergency of 2012 and the ongoing displacement of Syrians have all resulted in unsolicited donations and continue to highlight the need to control donations and minimize the inappropriate use of BMS in a more coordinated manner. While quantitative data, such as weight gain, may not be available as evidence that the baby tent program was successful, the experience in Haiti did provide knowledge and experience for IFE response in subsequent emergencies. Since 2010, IYCF counseling and support have become increasingly prominent components in emergencies, including in the Syrian crisis, where similar baby ‘caravans’ are being operated in refugee camps of Jordan and relactation is more successful. A scaled up interest in IFE has been noticed, including revisions of standard operating procedures for the handling of BMS, new assessment methodologies and development of appropriate and feasible indicators for assessment.

If the baby tent program with RUIF model is to be implemented as a component of IYCF emergency programs, specific actions must be taken by the IYCF in emergencies community. A generic protocol should be drafted and readily adapted to the context of emergencies which require IYCF interventions. This protocol should contain: admission and discharge criteria, standardized infant records forms and guidance on monitoring infant growth, minimal program monitoring and evaluation package, training package, appropriate numbers of staff, logistics components which address procurement, warehousing, transport and waste disposal issues. An IYCF toolkit encompassing these areas is underdevelopment. Training NGOs on the baby tent/RUIF model for IYCF in emergencies should be a mandatory part of preparedness activities. Special attention should be paid to NGOs with longstanding programs in emergency prone areas, and how short-term emergency IYCF programming can complement their programs. A stockpile of suitable and precise scales and length mats should be maintained for rapid deployment. Finally, it is important to remember that the use of BMS is only a small part of an overall IYCF program, albeit a very intensive one. It is easy to lose sight of the overall support for breastfeeding mothers given the added complexity and demands of the BMS component, but programs should still focus on the broader aspects of IYCF: support for breastfeeding mothers and appropriate complimentary feeding of older infants.
